# Can Antidepressants Prevent Pegylated Interferon-α/Ribavirin-Associated Depression in Patients with Chronic Hepatitis C: Meta-Analysis of Randomized, Double-Blind, Placebo-Controlled Trials?

**DOI:** 10.1371/journal.pone.0076799

**Published:** 2013-10-30

**Authors:** Xin-Jiang Hou, Jing-Hang Xu, Jun Wang, Yan-Yan Yu

**Affiliations:** Department of Infectious Diseases, Peking University First Hospital, Peking University, Beijing, People's Republic of China; Kaohsiung Medical University Hospital, Kaohsiung Medical University, Taiwan

## Abstract

**Background:**

Antidepressants are effective in treating interferon-α/ribavirin (IFN-α/RBV)-associated depression during or after treatment of chronic hepatitis C (CHC). Whether antidepressant prophylaxis is necessary in this population remains under debate.

**Methods:**

Comprehensive searches were performed in Medline, Embase, Cochrane Controlled Trials Register and PubMed. Reference lists were searched manually. The methodology was in accordance with the 2009 PRISMA (Preferred Reporting Items for Systematic Reviews and Meta-Analysis) Statement.

**Results:**

We identified six randomized, double-blind, placebo-controlled trials involving 522 CHC patients treated with pegylated (PEG)-IFN-α plus RBV. The antidepressants used were escitalopram, citalopram, and paroxetine, which are selective serotonin reuptake inhibitors (SSRIs). The rates of depression (17.9% vs. 31.0%, P = 0.0005), and rescue therapy (27.4% vs. 42.7%, P<0.0001) in the SSRI group were significantly lower than those in the placebo group. The rate of sustained virological response (SVR) (56.8% vs. 50.0%, P = 0.60) and drug discontinuation (18.7% vs. 21.1%, P = 0.63) in the SSRI group did not differ significantly to those in the placebo group. In terms of safety, the incidence of muscle and joint pain (40.8% vs. 52.4%, P = 0.03) and respiratory problems (29.3% vs. 40.1%, P = 0.03) were lower, but the incidence of dizziness was significantly higher (22.3% vs. 10.2%, P = 0.001) in the SSRI group.

**Conclusion:**

Prophylactic SSRI antidepressants can significantly reduce the incidence of PEG-IFN-α/RBV-associated depression in patients with CHC, with good safety and tolerability, without reduction of SVR.

## Introduction

Globally, hepatitis C virus (HCV) has become one of the leading causes of chronic liver diseases and affects >170 million people [1], [2]. The severity of the disease associated with HCV infection varies from asymptomatic chronic infection to all types of end-stage liver diseases, including cirrhosis and hepatocellular carcinoma [3]. To date, combination of pegylated interferon-α (PEG-IFN-α) plus ribavirin (RBV) is considered to be the most effective treatment for chronic hepatitis C (CHC). It is recommended in the American Association for the Study of Liver Diseases (AASLD) practical guidelines [4].

However, IFN-α-based therapy is associated with an approximately 70% incidence of mild to moderate depressive syndromes [5]–[7] and 20–40% incidence of major depression in HCV patients [7]. IFN-associated depression can lead to deterioration in quality of life, and has become a major contribution to treatment withdrawal, non-compliance, dose reduction of IFN/RBV, and even attempted suicide [8]–[15]; all of which can result in treatment failure [16]–[18]. Furthermore, depressive symptoms are closely related to poor virological response [19], [20]. Therefore, there is urgency in preventing the occurrence of depression in IFN-α-based therapy.

Although some studies have reported that antidepressants are effective in treating IFN-α/RBV-associated depression during or after treatment of CHC [21]–[29], whether antidepressant prophylaxis is necessary in this population remains a subject for debate [26]–[36].

Consequently, the aim of this meta-analysis was to evaluate the efficacy and safety of pretreatment with antidepressants to prevent IFN-α/RBV-associated depression in patients with CHC treated with antiviral therapy in randomized, double-blind, placebo-controlled trials.

## Materials and Methods

### Literature search

Two authors performed a literature search on Medline (1966 to March 2013), PubMed (updated to March 2013), Embase (1980 to March 2013), and Cochrane Controlled Trials Register (Cochrane Library Issue 1, 2013) using the keywords “hepatitis C AND interferon AND depression”. A manual search of publications and manual review of major journals in internal medicine, gastroenterology, hepatology, and infectious diseases (2000–2012) were also performed. This meta-analysis was conducted and reported according to the PRISMA (Preferred Reporting Items for Systematic Reviews and Meta-Analysis) Statement issued in 2009 [37].

### Inclusion/exclusion criteria

Inclusion criteria were: (1) study population with CHC; (2) antiviral regime was IFN-<alpha>/RBV combination therapy; (3) studies that reported data for outcomes at the end of treatment and follow-up period; (4) studies with full text; and (5) English studies. Exclusion criteria were: (1) non-randomized controlled trials (RCTs); (2) non-prophylaxis studies with pretreatment with antidepressants before antiviral therapy; (3) studies about human immunodeficiency virus, hepatitis B virus or other virus co-infections; and (4) studies about organ transplantation.

### Data extraction

Two investigators independently evaluated each study, each of whom was blinded to the other. Any discrepancy was resolved through discussion. Extracted information included study characteristics (first author, study location, and year); patient baseline characteristics (sex, risk factors, baseline HCV RNA, HCV genotype, body mass index, and percentage with fibrosis and cirrhosis); and antidepressant and antiviral regimens. Outcomes included: total number of patients; number of patients with IFN-associated depression at the end of treatment; number of patients with sustained virological response (SVR) at the end of follow-up (defined as undetectable HCV RNA at the end of follow-up); total number of patients with treatment discontinuation; number of patients with serious adverse events; number of patients with dose reduction due to adverse events; and number of patients who received antidepressant rescue treatment.

### Quality of methodology

Assessment of the methodological quality of the trials was based on the Jadad composite scale [38], which evaluated randomization, concealment and reporting of patient withdrawal and dropout rates, with ≥3 scores defined as high quality, and ≤2 as low quality. Heterogeneity was assessed for each analysis. The methodological quality was assessed independently by two of the authors.

### Statistical methods

The statistical analyses were conducted and Forest plots were generated using RevMan 5.2.3 (Nordic Cochrane Center, Rigshospitalet, Copenhagen, Denmark). The primary endpoints were the rate of incidence of depression and the rate of SVR. The secondary endpoints were the rate of drug discontinuation or withdrawal, rate of antidepressant rescue treatment, and the rate of adverse events. For the calculation of risk ratios (RRs), patients assigned to prophylactic antidepressant treatment were compared with those assigned to placebo in the same trial. The RRs were calculated along with their respective 95% confidence intervals (CIs) and presented for each individual study. Statistical heterogeneity between trials was evaluated by the χ^2^ and I^2^ tests, with significance being set at P<0.10. In the absence of significant heterogeneity (P>0.10), the fixed-effect method was used to combine the results. When heterogeneity was confirmed (P≤0.10), the random-effect method was used, and sensitivity analyses and meta-regressions were performed with study characteristics to evaluate potential sources of heterogeneity. Stratified analyses were performed using the variables found to be statistically significant in the meta-regressions. Any potential publication bias was evaluated by inspecting funnel plots and the Begg–Mazumdar test [39].

## Results

### Search results

**Figure 1:**
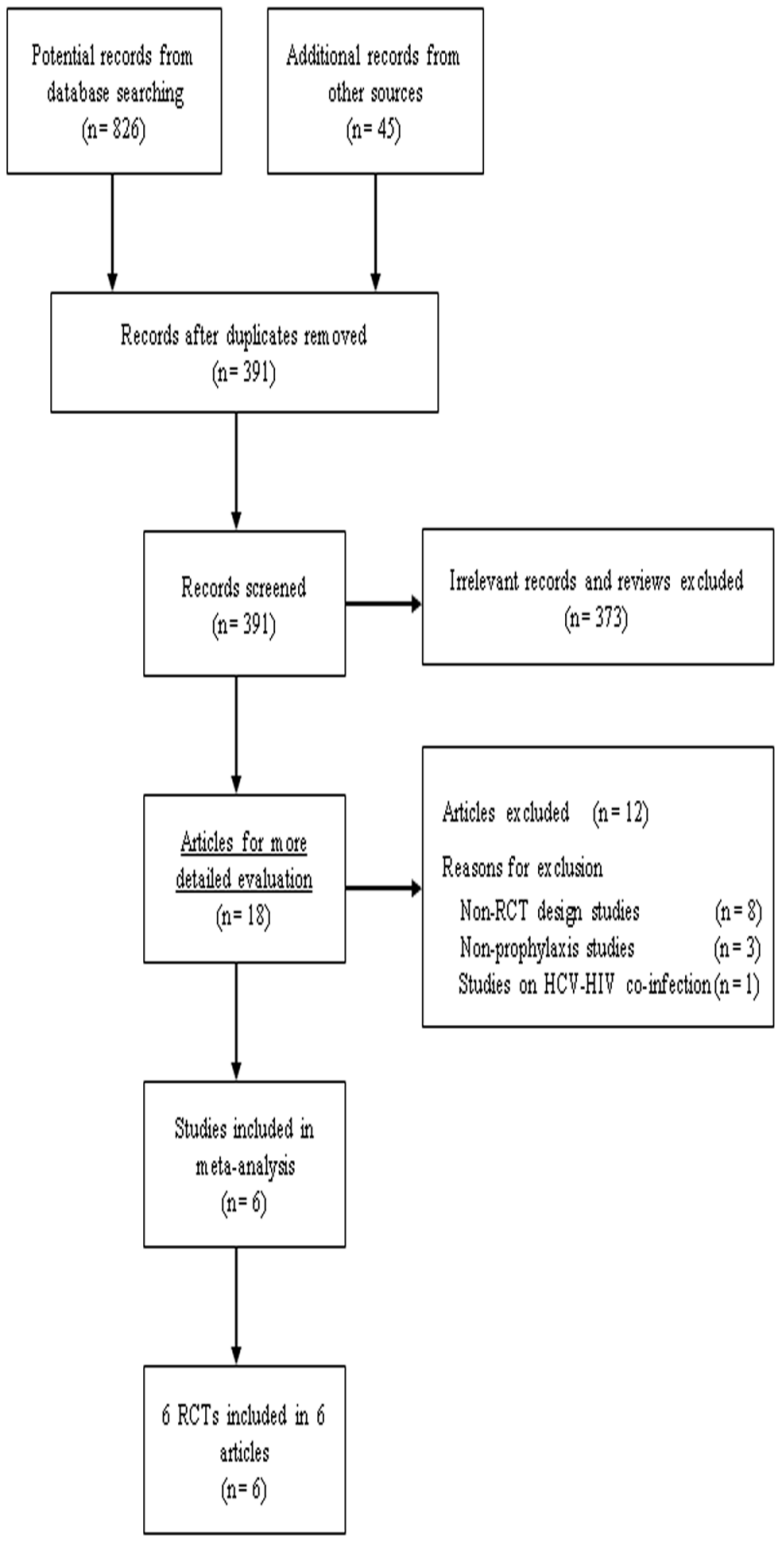
Flow diagram of literature search and selection process.

Of the 871 articles found after initial searching, six studies involving 522 patients met the inclusion criteria [29]–[34]. A flow chart summarizing the search and screening process is presented in Figure 1. Placebo-controlled design and intention-to-treat analysis were conducted in all trials. The antidepressants used in the present meta-analysis were escitalopram, citalopram, and paroxetine, all of which are selective serotonin reuptake inhibitors (SSRIs). The antiviral therapies administrated in all studies were PEG-IFN-α/RBV, with exception of IFN-α/RBV in some patients in two studies. The baseline patient characteristics and depression scoring system are listed in Table 1. Study design and antidepressant and antiviral regimens are listed in Table 2.

**Table 1:** Basic Characteristics and Depression Assessing System Applied in Trials Included in the Meta-Analysis.

Study	Status of CHC	Status of Depression	Measures for Depression Assessment
Morasco BJ [29]	>18 years old, eligible for IFN/RBV therapy.	Exclusion criteria: hypersensitivity to paroxetine, active delirium, current major depressive disorder, bipolar disorder, a schizophrenia-spectrum disorder, and a substance use disorder within the last six months.	DSM-IV (SCID) HAM-D
Raison CL [30]	18–65 years old, Exclusion criteria: other liver disease, unstable cardiovascular, endocrinologic, haematologic, renal or neurologic disease.	Exclusion criteria: history of schizophrenia or bipolar disorder, diagnosis of major depression or substance, abuse/dependence within 6 months of study entry.	SCID MADRS
Diez-Quevedo C [31]	18–65 years old, ≥16 months of persistent elevated ALT. Exclusion criteria: other liver disease, unstable cardiovascular, endocrinologic, haematologic, renal, autoimmune, neurologic disease, Co-infection with HBV, HIV, neutrophil count<1500,platelet count <70,000 per µl, hemoglobin<12 g/dl in men and <11 g/dl in women, non reliable contraception in women.	Exclusion criteria: history or current diagnosis of schizophrenia or bipolar disorder, dementia; the presence, within 2 months of study entry, of drug, or alcohol abuse, symptomatic mental disorders(including major depressive disorders, dysthymia, and anxiety disorders other than specific phobias),the use of any psychiatric medication(patients were only allowed to maintain treatments with benzodiazepines at constant dosages). Zolpidem was used for insomnia when needed.	DSM-IV
De Knegt RJ [32]	18–70 years old, Exclusion criteria: presence of contra-indications, for anti-viral therapy, abnormal values for thyroid stimulating hormone.	Exclusion criteria: a concurrent psychiatric axis I diagnosis, concurrent use of psychotropic drugs, a history or other evidence of severe illness or malignancy and any other condition which would make the patient unsuitable for the study.	M.I.N.I. DSM-IV MADRS BDI SCL-90
Morasco BJ [33]	≥18 years old, eligible for antiviral therapy.	Exclusion criteria: ongoing depression or active psychotic symptoms during the previous 3 months, substance abuse in the previous 6 months, medical comorbidities that could interfere with treatment, or current antidepressant use.	DSM-IV (SCID) MADRS
Schaefer M [34]	>18 years old, treatment-naive patients, serum HCV RNA levels ≥1000 IU/mL. Exclusion criteria: pretreatment with IFN or immunotherapy, other chronic infection, or an autoimmune or severe somatic comorbid condition.	Exclusion criteria: a lifetime diagnosis of an affective disorder, drug abuse in the past 12 months, treatment with antidepressants during the past 3 years, or a history of any other axis I disorder.	DSM-IV (SCID) MADRS

HBV: Heptatitis B virus, HIV: Human Immunodeficiency Virus, DSM-IV: Diagnostic and Statistical Manual of Mental Disorders, Fourth Edition, M.I.N.I: Mini-international Neuropsychiatric Interview, SCID: Structured Clinical Interview for DSM–IV; MADRS: Montgomery–Asberg Depression Rating Scale, BDI: Beck Depression Inventory, SCL-90:Symptom Check List-90.

**Table 2:** Characteristic of Randomized Controlled Trials Included in the Meta-Analysis.

Study	Study design	SSRI	Anti depression regime	Antiviral regime	Sample size (AD/control)	Quality Score
Morasco BJ [29]	multi-center, double-blind, placebo-controlled.	Paroxetine	4 weeks of prophylaxis, subsequent 24–48weeks of administration.	genotype-based dose of PEG-IFN-α2a,2b or IFN-α, weight-based dose of RBV.	14/19	4
Raison CL [30]	multi-center, double-blind, placebo-controlled.	Paroxetine	2 weeks of prophylaxis, subsequent 24 weeks of administration.	PEG-IFN-2α, 2b or IFN-α, RBV according to treating Physicians.	28/33	4
Diez-Quevedo C [31]	multi-center, double-blind, placebo-controlled.	Escitalopram	2 weeks of prophylaxis, subsequent 14 weeks of administration.	PEG-IFN-α2a,180 mg/w, RBV 800–1200 mg.	66/63	4
De Knegt RJ [32]	multi-center, double-blind, placebo-controlled.	Escitalopram	2 weeks of prophylaxis, subsequent 24 weeks of administration.	genotype-based dose of PEG-IFN-α2a, weight-based dose of RBV.	40/39	4
Morasco BJ [33]	double-center, double-blind, placebo-controlled.	Citalopram	2 weeks of prophylaxis, subsequent 24–48weeks of administration.	genotype-based dose of PEG-IFN-α. guideline-based dose of RBV.	19/20	4
Schaefer M [34]	multi-center, double-blind, placebo-controlled.	Escitalopram	2 weeks of prophylaxis, subsequent 24–48weeks of administration.	genotype-based dose of PEG-IFN-α2a,weight-based dose of RBV.	90/91	5

SSRI: selective serotonin reuptake inhibitor; study quality was evaluated on the 7-item Jadad scale, with a range of 0–5.

### Rate of depression

**Figure 2:**
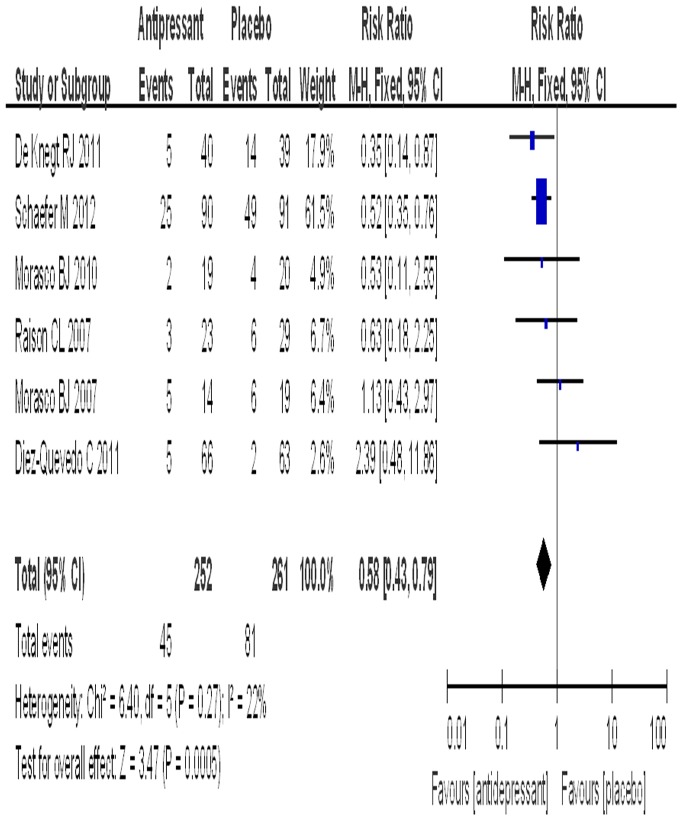
The effect of antidepressant on the incidence of depression.

All six trials provided data about the rate of PEG-IFN-α/RBV-associated depression. The SSRI group included 252 patients with a rate of depression of 17.9%, while the placebo group included 261 patients with a rate of depression of 31.0%. The test for heterogeneity of the data obtained a P value of 0.27, thus the hypothesis of homogeneity was achieved. An estimated RR of 0.58 (95% CI: 0.43–0.79) was obtained after the data were pooled, and a significant effect on the rate of depression was found for the prophylaxis of SSRIs to prevent PEG-IFN-α/RBV-associated depression in patients with CHC (P = 0.0005) (Figure 2). The subgroup analyses of different SSRIs for the rate of depression are shown in Table 3.

**Table 3:** Subgroup Analysis with Respect to Antidepressants for Different End-Points.

End-points	No. of patients with available data	SSRI group (No./%)	Placebo group (No./%)	P for homogeneity/Model	Exact RR (95%CI)	P
Rate of depression	428/Escitalopram and citalopram	37/17.2	69/32.4	0.24/Fixed	0.54 [0.38–0.75]	0.0003
	389/Escitalopram	35/17.9	65/33.7	0.12/Fixed	0.54 [0.38–0.76]	0.0004
	94/Paroxetine	8/19.0	14/26.9	0.23/Fixed	0.72 [0.34–1.54]	0.40
	474/Escitalopram and Paroxetine	43/18.5	77/32.0	0.17/Fixed	0.58 [0.43–0.80]	0.0007
Rate of SVR	327/Escitalopram andCitalopram	93/57.4	90/54.5	0.34/Fixed	1.05 [0.87–1.27]	0.60
	288/Escitalopram	86/59.4	80/55.2	0.25/Fixed	1.09 [0.90–1.33 ]	0.38
	321/Escitalopram and Paroxetine	93/59.2	82/50.0	0.05/Random	1.20 [0.80–1.79]	0.39
Rate of discontinuation	428/Escitalopram and Citalopram	34/15.8	30/14.1	0.28/Fixed	1.12 [0.71–1.77]	0.61
	388/Escitalopram	31	25	0.22/Fixed	1.22 [0.75–1.99]	0.43
	94/Paroxetine	14/33.3	26/50.0	0.71/Fixed	0.66 [0.40–1.09]	0.10
	483/Escitalopram and Paroxetine	45/18.9	51/20.8	0.19/Fixed	0.95 [0.67–1.35]	0.78
Rate of reception of rescue therapy	389/Escitalopram	56/28.6	87/45.1	0.05/Random	0.62 [0.50–0.77]	<0.0001

SSRI: selective serotonin reuptake inhibitor.

### Rate of SVR

**Figure 3:**
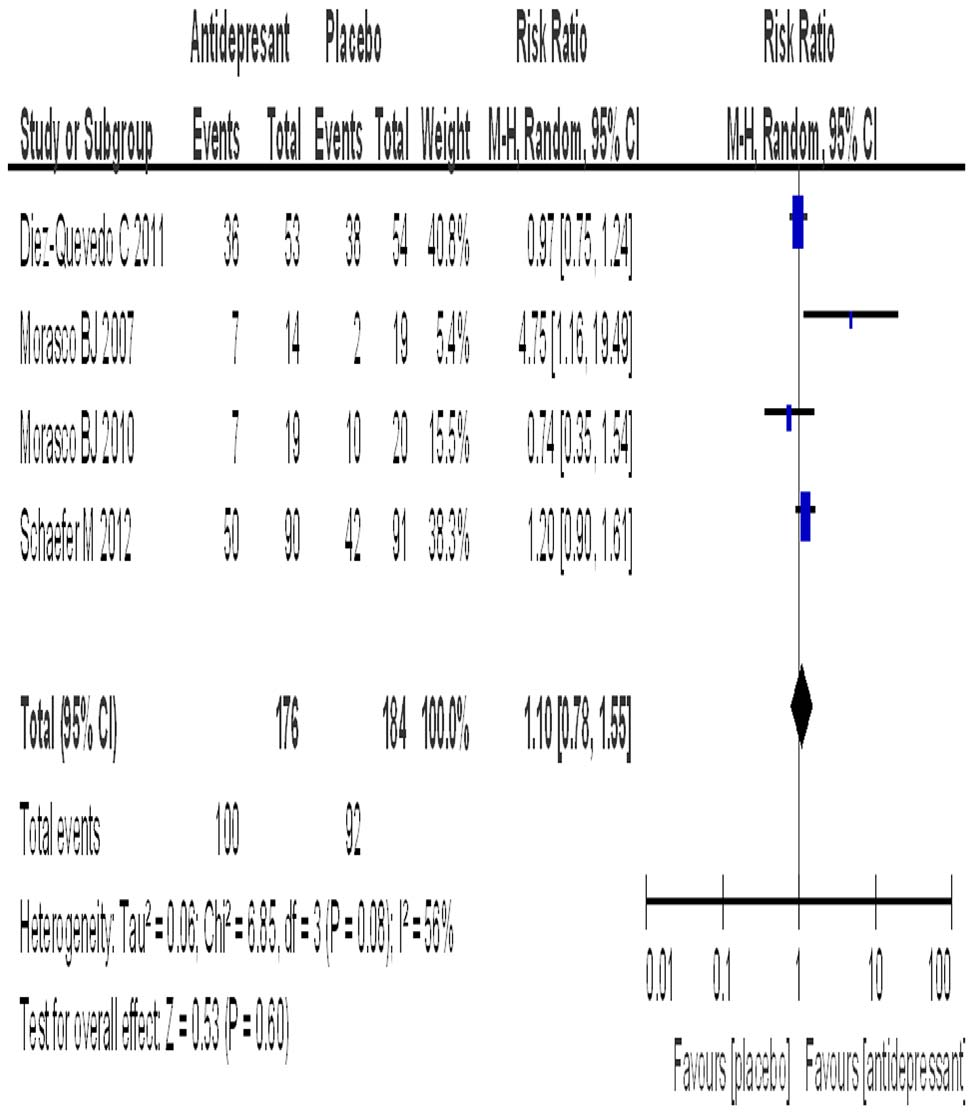
The effect of antidepressant on the SVR.

Four trials reported data on the effect of antidepressants on SVR. The SSRI group included 176 patients with a rate of SVR of 56.8%, while the placebo group included 184 patients with a rate of SVR of 50.0%. The test for the heterogeneity of the data obtained a P value of 0.08, thus the random model was used. An estimated RR of 1.10 (95% CI: 0.78–1.55) was obtained after the data were pooled. Antidepressant prophylaxis had no significant effect on the rate of SVR in patients with CHC (P = 0.60) (Figure 3). The subgroup analyses of different SSRIs for the rate of SVR are shown in Table 3.

### Rate of drug discontinuation

**Figure 4:**
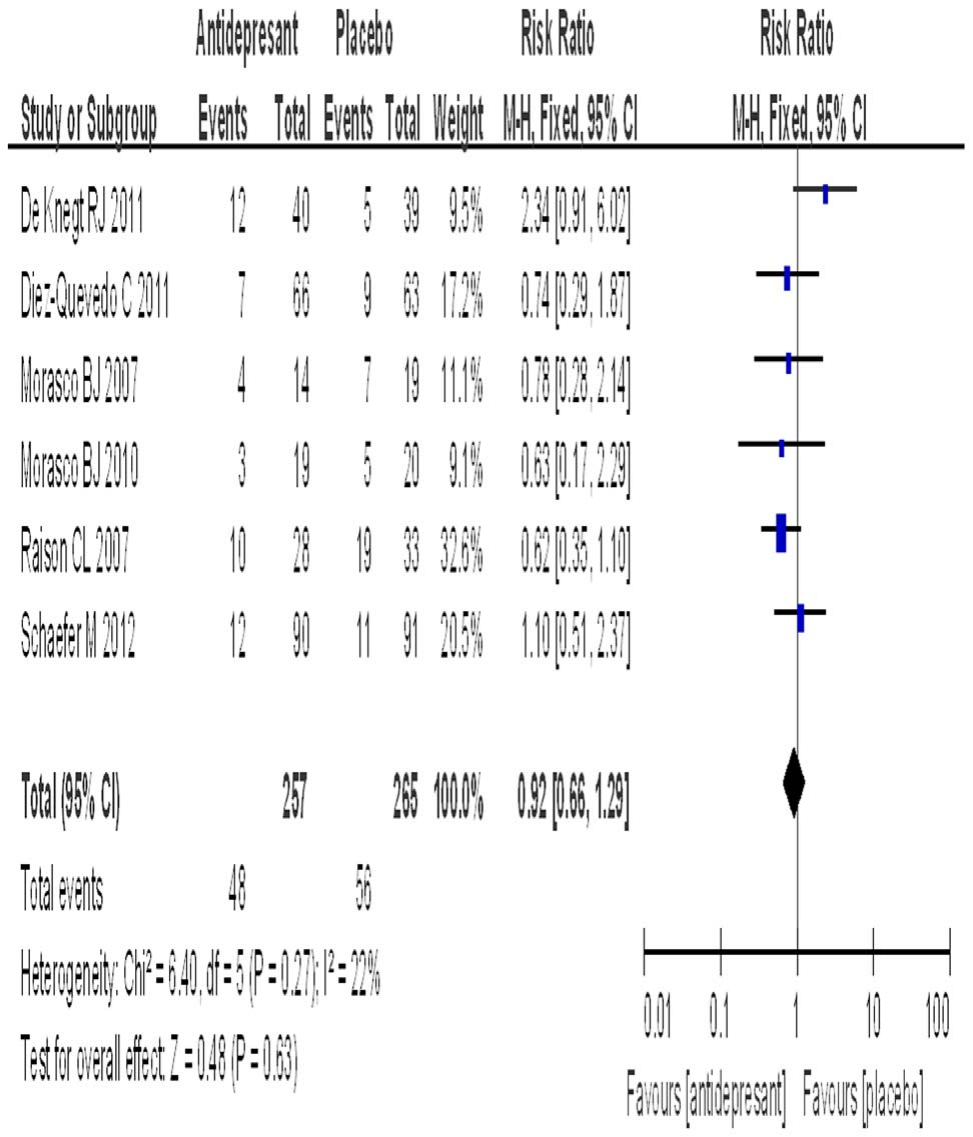
The comparison of discontinuation.

All of the trials reported data on drug discontinuation. The SSRI group included 257 patients with a rate of discontinuation of 18.7%, while the placebo group included 265 patients with a rate of discontinuation of 21.1%. The test for heterogeneity of the data obtained a P value of 0.27, thus the hypothesis of homogeneity was achieved. An estimated RR of 0.92 (95% CI: 0.66–1.29) was obtained after the data were pooled. Antidepressant prophylaxis had no significant effect on the rate of discontinuation in patients with CHC (P = 0.63) (Figure 4). The subgroup analyses of different SSRIs for the rate of discontinuation are shown in Table 3.

### Rate of rescue therapy

**Figure 5:**
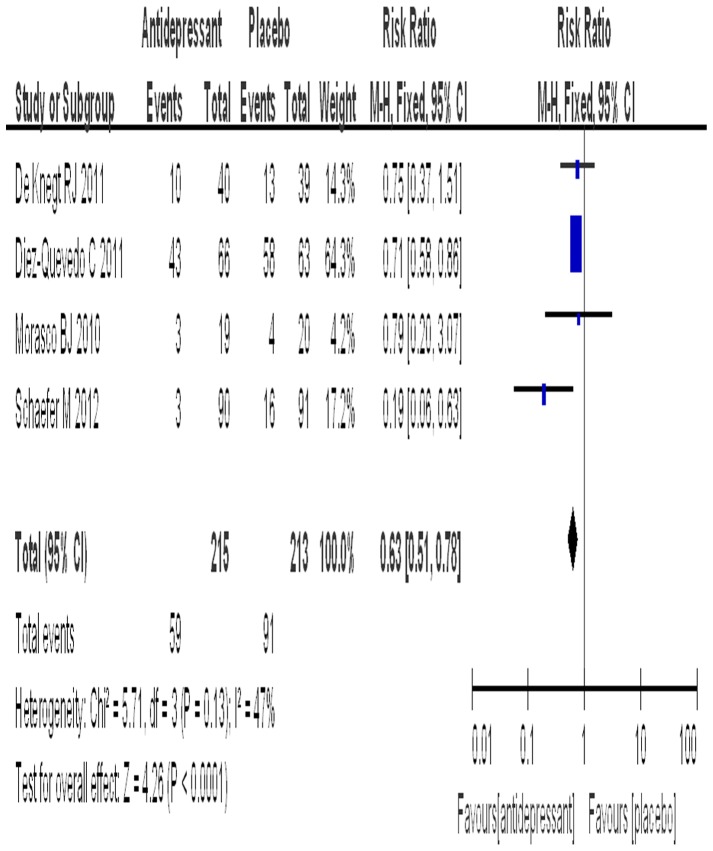
The comparison of reception of rescue therapy.

Four trials reported data on the number of patients receiving rescue therapy. The SSRI group included 215 patients with a rate of rescue therapy of 27.4%, while the placebo group included 213 patients with a rate of rescue therapy of 42.7%. The test for heterogeneity of the data obtained a P value of 0.13, thus the hypothesis of homogeneity was achieved. An estimated RR of 0.63 (95% CI: 0.51–0.78) was obtained after the data were pooled, and a significant effect on the rate of rescue therapy was found (P<0.0001) (Figure 5). The subgroup analyses of different SSRIs for the rate of rescue therapy are shown in Table 3.

### Adverse events

**Figure 6:**
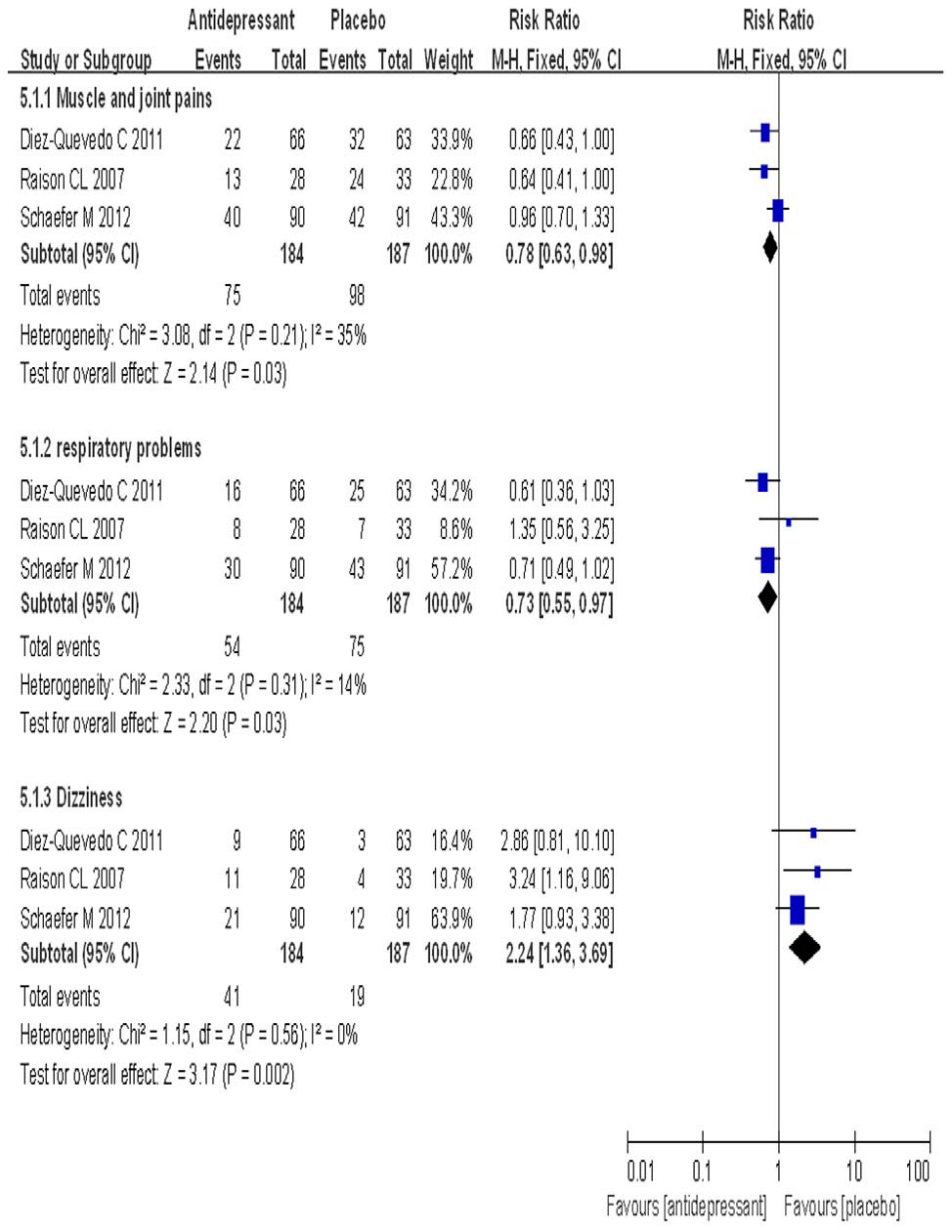
The comparison of three adverse events.

Data for adverse events were extracted from three trials and subjected to meta-analysis (Table 4). The rate of muscle and joint pain in the SSRI group was significantly lower than that in the placebo group (40.8% vs. 52.4%, P = 0.03). The rate of respiratory problems in the SSRI group was significantly lower than that in the placebo group (29.3% vs. 40.1%, P = 0.03) (Figure 6). The rate of respiratory problems in the escitalopram group was also significantly lower than that in the placebo group (29.5% vs. 44.2%, P = 0.008) (Table 4). However, the rate of dizziness in the SSRI group was significantly higher than that in the placebo group (22.3% vs. 10.2%, P = 0.001) (Figure 6). The rate of dizziness in the escitalopram group was also significantly higher than that in the placebo group (19.2% vs. 9.7%, P = 0.02) (Table 4).

**Table 4:** Summary of Common Adverse Events associated the Antidepressants and Antiviral Treatment.

Adverse events	No. of patients with available data	SSRI group (No./%)	Placebo group (No./%)	Exact RR (95% CI)	*P*	*P* for Homogeneity
Muscle and joint pain	371(Escitalopram+ Paroxetine)	75/40.8	98/52.4	0.63 [0.42, 0.96]	0.03	0.15
	310(Escitalopram)	62/39.7	74/48.1	0.72 [0.46, 1.12]	0.14	0.15
Dizzness	371(Escitalopram+ Paroxetine)	41/22.3	19/10.2	2.65 [1.46, 4.80]	0.001	0.52
	310(Escitalopram)	30/19.2	15/9.7	2.26 [1.16, 4.43]	0.02	0.57
Headache	371(Escitalopram+ Paroxetine)	61/33.2	72/38.5	0.81 [0.53, 1.24]	0.33	0.56
	310(Escitalopram)	49/31.4	52/33.8	0.90 [0.56, 1.45]	0.67	0.90
Fatigue	371(Escitalopram+ Paroxetine)	92/50.0	102/54.5	0.83 [0.56, 1.25]	0.38	0.28
	310(Escitalopram)	76/48.7	87/56.5	0.73 [0.47, 1.15]	0.17	0.42
Sexual dysfunction	371(Escitalopram+ Paroxetine)	17/9.2	8/4.3	2.35 [0.98, 5.65]	0.06	0.83
	310(Escitalopram)	13/8.3	5/3.2	2.70 [0.94, 7.82]	0.07	0.71
Loss of appetite	371(Escitalopram+ Paroxetine)	37/20.1	36/19.3	1.09 [0.65, 1.82]	0.75	0.09
	310(Escitalopram)	24/15.4	27/17.5	0.85 [0.47, 1.55]	0.60	0.12
Sleep disturbance	371(Escitalopram+ Paroxetine)	71/38.6	85/45.5	0.76 [0.50, 1.15]	0.19	0.25
	310(Escitalopram)	57/36.5	71/46.1	0.68 [0.43, 1.07]	0.09	0.26
Influenza-like illness	371(Escitalopram+ Paroxetine)	54/29.3	64/34.2	0.81 [0.51, 1.27]	0.35	0.58
	310(Escitalopram)	42/26.9	52/33.8	0.72 [0.43, 1.19]	0.20	0.87
Skin problems	371 (Escitalopram+ Paroxetine)	94/51.1	102/54.5	0.83 [0.54, 1.29]	0.42	0.30
	310(Escitalopram)	89/57.1	92/59.7	0.90 [0.57, 1.44]	0.67	0.20
Respiratory problems	371(Escitalopram+ Paroxetine)	54/29.3	75/40.1	0.61 [0.40, 0.94]	0.03	0.27
	310(Escitalopram)	46/29.5	68/44.2	0.53 [0.33, 0.85]	0.008	0.78
Hair loss	371(Escitalopram+ Paroxetine)	27/14.7	33/17.6	0.80 [0.46, 1.40]	0.43	0.38
	310(Escitalopram)	24/15.4	29/18.8	0.79 [0.43, 1.43]	0.44	0.17
Anemia	310(Escitalopram)	23/14.7	22/14.3	1.05 [0.56, 1.97]	0.89	0.21
Irritability	310(Escitalopram)	16/10.2	26/16.9	0.55 [0.28, 1.08]	0.08	0.68
nausea or vomiting	371(Escitalopram+ Paroxetine)	66/35.9	69/36.9	0.97 [0.63, 1.48]	0.88	0.32
	310(Escitalopram)	51/32.7	56/36.4	0.85 [0.53, 1.36]	0.50	0.42
Gastrointestinal illness	371(Escitalopram+ Paroxetine)	45/24.5	33/17.6	1.55 [0.93, 2.58]	0.09	0.87
	310(Escitalopram)	35/22.4	24/15.6	1.57 [0.88, 2.79]	0.12	0.60

SSRI: selective serotonin reuptake inhibitor. Escitalopram+ Paroxetine indicated that one of these two SSRIs was used in one trial and the data on the two SSRIs were meta-analyzed. Escitalopram indicated that data about the antidepressant were meta-analyzed.

## Discussion

We performed this meta-analysis of randomized double-blind placebo-controlled clinical trials to evaluate the efficacy and safety of prophylactic antidepressants to prevent PEG-IFN-α/RBV-associated depression in patients with CHC. The results reveal that preemptive and concomitant therapy with SSRIs can significantly reduce the incidence of PEG-IFN-α/RBV-associated depression in patients with CHC. SSRI interventions were safe, did not negatively decrease SVR, and did not influence the rate of drug discontinuation or withdrawal.

Interferon-α/RBV-associated depression is characteristic of a serotonergic deficiency and changes in the serotonin signaling pathway, which requires the prophylactic administration of SSRIs [34], because these drugs increase serotonin and have been widely used over the past decade, with a good safety profile [40].

The pooled rate of depression in the placebo group was 31.0%, which fell within the 20–40% incidence of depression in patients treated with IFN-α/RBV [7]. Compared with placebo, prophylactic antidepressants can significantly reduce the incidence of PEG-IFN-α/RBV-associated depression, by up to 18.7% in CHC patients without a history of severe mental disease. Although standard INF-α was used in some CHC patients in two studies [29], [30], the results from the other four trials showed that antidepressants markedly decreased the incidence of depression to 17.2% during PEG-IFN-α-based antiviral therapy. Furthermore, both current types of PEG-IFN-α, that is, PEG-IFN-α2a and 2b, were administrated in all six trials, so the type of IFN-α may be ignored when prophylactic SSRIs are used in patients with CHC. Escitalopram and paroxetine share the same mechanism of action to treat depression, although the former is superior to the latter for prophylactic intervention according to the present study. Escitalopram was developed after citalopram and is considered safer and more effective for treating major depression. However, a previous meta-analysis showed that escitalopram was not superior to citalopram in short-to-medium term treatment of major depressive disorder [41]. The rate of depression was 10.5% when citalopram was used [33], which was lower than 17.9%—the pooled rate of depression in three trials when escitalopram was used [31], [32], [34]. However, the sample size in the trial on citalopram was only 39 patients, which was 10% of the pooled sample in the three trials of escitalopram. Clinically, clinicians should pay more attention to the results of meta-analyses.

With respect to the effect of SSRIs on the SVR, the results varied among the trials, although the trials were of similar design [29], [31], [33], [34]. The pooled SVR rate in the setting of prophylactic SSRIs did not differ significantly compared with that in the placebo group. Subgroup analysis also reached a similar conclusion. Consequently, prophylactic SSRIs have little effect on the SVR in patients with CHC when they are treated with antiviral therapy. However, we suggest that escitalopram should be used because of the higher SVR rate and its increased use for intervention in depression.

IFN-α/RBV-associated depression was an important reason for discontinuing antiviral therapy [42], [43]. Consequently, successful treatment of CHC needs prevention of depression. The pooled rate of drug discontinuation differed significantly between the SSRI and placebo groups after subgroup analyses, which was consistent with the results from each trial individually. These results suggest that prophylactic SSRIs have little effect on drug discontinuation during treatment of CHC with the currently available antiviral therapy.

The lower rates of rescue therapy, muscle and joint pain and respiratory problems in the SSRI group indicated the better safety and tolerability of SSRIs. These results suggest that prophylactic SSRIs reduce the occurrence of these two adverse events associated with PEG-IFN-α/RBV combination therapy in patients with CHC. However, the pooled rate of dizziness was higher in the SSRI group than the placebo group, which was consistent with the results of each separate trial. This reminds clinicians to pay more attention to the occurrence of this adverse event when prophylactic SSRIs are administrated to patients with CHC. Therefore, prophylactic SSRIs did not worsen most adverse events apart from dizziness. Moreover, adverse events (e.g. diarrhea, headache, and sleep disturbance) were not increased as a result of concurrent administration of antiviral therapy and SSRIs. Although it was reported that citalopram could be safely used during IFN treatment in patients with CHC [44], there were risks of using SSRIs during IFN treatment, because CHC may alter the pharmacokinetics of SSRIs. Therefore, SSRI dose may require adjustment to optimize the treatment [40].

We need to emphasize that according to our clinical experience and inclusion criteria, the optimal candidates for SSRI prophylaxis are CHC patients with concurrent subsyndromal depression symptoms (such as insomnia and anxiety) or a history of past major depressive disorders and eligibility for PEG-IFN-α/RBV treatment.

There were several limitations to our study. First, only six eligible RCTs were included, and we were limited by data availability. Second, sample sizes in most of the trials were small and included <50 subjects. Third, the possibility of omission of unpublished or ongoing studies still exist, although publication bias was not found according to the funnel plots and the Begg–Mazumdar test. Fourth, possible variation in the definition of depression and severity of depression were present in the studies included in our analysis, although the criteria for diagnosis and severity of depression were similar in all studies. These defects may weaken our results. Further analysis of increased numbers of multicenter RCTs with larger sample sizes are warranted to evaluate comprehensively the true value of prophylactic SSRIs to prevent PEG-IFN-α/RBV-associated depression during antiviral therapy in patients with CHC.

Despite these limitations, our meta-analysis of double-blind, placebo-controlled RCTs shows that prophylactic SSRIs can significantly reduce the incidence of PEG-IFN-α/RBV-associated depression in patients with CHC, with good safety and tolerability and without reduction of SVR.

## Supporting Information

Figure S1 **PRISMA 2009 Flow Diagram.**
(DOC)

Checklist S1 **PRISMA 2009 Checklist.**
(DOC)

## References

[1] Shepard CW, Finelli L, Alter MJ (2005) Global epidemiology of hepatitis C virus infection. Lancet Infect Dis 5: 558–67. 16122679 10.1016/S1473-3099(05)70216-4

[2] Lavanchy D (2009) The global burden of hepatitis C. Liver Int 29: 74–81. 10.1111/j.1478-3231.2008.01934.x 19207969

[3] Perz JF, Armstrong GL, Farrington LA, Hutin YJ, Bell BP (2006) The contributions of hepatitis B virus and hepatitis C virus infections to cirrhosis and primary liver cancer worldwide. J Hepatol 45: 529–38. 16879891 10.1016/j.jhep.2006.05.013

[4] Ghany MG, Strader DB, Thomas DL, Seeff LB (2009) Diagnosis, management, and treatment of hepatitis C: an update. Hepatology 49: 1335–74. 19330875 10.1002/hep.22759 PMC7477893

[5] Reichenberg A, Gorman JM, Dieterich DT (2005) Interferon-induced depression and cognitive impairment in hepatitis C virus patients: a 72 week prospective study. AIDS Suppl 3: S174–8. 10.1097/01.aids.0000192087.64432.ae 16251815

[6] Schaefer M, Schwaiger M, Garkisch AS, Pich M, Hinzpeter A, et al (2005) Prevention of interferon-alpha associated depression in psychiatric risk patients with chronic hepatitis C. J Hepatol 42: 793–8. 15885349 10.1016/j.jhep.2005.01.020

[7] Schäfer A, Wittchen HU, Seufert J, Kraus MR (2007) Methodological approaches in the assessment of interferon-alfa-induced depression in patients with chronic hepatitis C-a critical review. Int J Methods Psychiatr Res 16: 186–201. 18188838 10.1002/mpr.229 PMC6878515

[8] Kraus MR, Schafer A, Csef H, Faller H, Mork H, et al (2001) Compliance with therapy in patients with chronic hepatitis C: associations with psychiatric symptoms, interpersonal problems, and mode of acquisition. Digest Dis Sci 46: 2060–5. 11680576 10.1023/a:1011973823032

[9] Zdilar D, Franco-Bronson K, Buchler N, Locala JA, Younossi ZM (2000) Hepatitis C, interferon alfa, and depression. Hepatology 31: 1207–11. 10827143 10.1053/jhep.2000.7880

[10] Sockalingam S, Links PS, Abbey SE (2011) Suicide risk in hepatitis C and during interferon-alpha therapy: a review and clinical update. J Viral Hepat 18: 153–60. 21070503 10.1111/j.1365-2893.2010.01393.x

[11] Cacoub P, Ratziu V, Myers RP, Ghillani P, Piette JC, et al (2002) Impact of treatment on extra hepatic manifestations in patients with chronic hepatitis C. J Hepatol 36: 812–8. 12044533 10.1016/s0168-8278(02)00067-3

[12] Bernstein D, Kleinman L, Barker CM, Revicki DA, Green J (2002) Relationship of health-related quality of life to treatment adherence and sustained response in chronic hepatitis C patients. J Hepatol 35: 704–8. 10.1053/jhep.2002.31311 11870387

[13] Schaefer M, Engelbrecht MA, Gut O, Fiebich BL, Bauer J, et al (2002) Interferon alpha (IFNa) and psychiatric syndromes: a review. Pro Neuro-Psychopharmacol Biol Psychiatry 26: 731–46. 10.1016/s0278-5846(01)00324-4 12188106

[14] Dieperink E, Willenbring M, Ho SB (2000) Neuropsychiatric symptoms associated with hepatitis C and interferon alpha: a review. Am J Psychiatry 157: 867–76. 10831463 10.1176/appi.ajp.157.6.867

[15] Guadagnino VA, Trotta MP, Carioti J, Caroleo B, Antinori A, et al (2006) Does depression symptomatology affect medication compliance during the first weeks of anti-HCV therapy in intravenous drug users? Digest Liver Dis 38: 119–24. 10.1016/j.dld.2005.10.008 16297672

[16] McHutchison JG, Manns M, Patel K, Poynard T, Lindsay KL, et al (2002) Adherence to combination therapy enhances sustained response in genotype-1-infected patients with chronic hepatitis C. Gastroenterol 123: 1061–9. 10.1053/gast.2002.35950 12360468

[17] Davis GL, Wong JB, McHutchison JG, Manns MP, Harvey J, et al (2003) Early virologic response to treatment with peginterferon alfa-2b plus ribavirin in patients with chronic hepatitis C. Hepatology 38: 645–52. 12939591 10.1053/jhep.2003.50364

[18] Shiffman ML, Di Bisceglie AM, Lindsay KL, Morishima C, Wright EC, et al (2004) Peginterferon alfa-2a and ribavirin in patients with chronic hepatitis C who have failed prior treatment. Gastroenterol 126: 1015–23. 10.1053/j.gastro.2004.01.014 15057741

[19] Raison CL, Broadwell SD, Borisov AS, Manatunga AK, Capuron L, et al (2005) Depressive symptoms and viral clearance in patients receiving interferon-alpha and ribavirin for hepatitis C. Brain Behav Immun 19: 23–7. 15581735 10.1016/j.bbi.2004.05.001

[20] Maddock C, Landau S, Barry K, Maulayah P, Hotopf M, et al (2005) Psychopathological symptoms during interferon-alpha and ribavirin treatment: effects on virologic response. Mol Psychiatry 10: 332–3. 15655564 10.1038/sj.mp.4001634

[21] Hauser P, Khosla J, Aurora H, Laurin J, Kling MA, et al (2002) A prospective study of the incidence and open-label treatment of interferon-induced major depressive disorder in patients with hepatitis C. Mol Psychiatry 7: 942–7. 12399946 10.1038/sj.mp.4001119

[22] Kraus MR, Schafer A, Faller H, Csef H, Scheurlen M (2002) Paroxetine for the treatment of interferon-alpha-induced depression in chronic hepatitis C. Aliment Pharmacol Therap 16: 1091–9. 12030950 10.1046/j.1365-2036.2002.01265.x

[23] Schaefer M, Schwaiger M, Garkisch AS, Pich M, Hinzpeter A, et al (2005) Prevention of interferon-alpha associated depression in psychiatric risk patients with chronic hepatitis C. J Hepatol 42: 793–8. 15885349 10.1016/j.jhep.2005.01.020

[24] Farah A (2002) Interferon-induced depression treated with citalopram. J Clin Psychiatry 63: 166–7. 10.4088/jcp.v63n0213c 11874221

[25] Gleason OC, Yates WR (1999) Five cases of interferon-alpha-induced depression treated with antidepressant therapy. Psychosom 40: 510–2. 10.1016/S0033-3182(99)71190-4 10581980

[26] Gleason OC, Yates WR, Isbell MD, Philipsen MA (2002) An open-label trial of citalopram for major depression in patients with hepatitis C. J Clin Psychiatry 63: 194–8. 11926717 10.4088/jcp.v63n0304

[27] Schramm TM, Lawford BR, MacdonaldGA, Cooksley WG (2000) Sertraline treatment of interferon-alfa-induced depressive disorder. Med J Aust 173: 359–61. 11062791 10.5694/j.1326-5377.2000.tb125687.x

[28] Kraus MR, Schäfer A, Schöttker K, Keicher C, Weissbrich B, et al (2008) Therapy of interferon induced depression in chronic hepatitis C with citalopram: a randomised, double-blind, placebo-controlled study. Gut 57: 531–6. 18079286 10.1136/gut.2007.131607

[29] Morasco BJ, Rifai MA, Loftis JM, Indest DW, Moles JK, et al (2007) A randomized trial of paroxetine to prevent interferon-alpha-induced depression in patients with hepatitis C. J Affect Disord 103: 83–90. 17292481 10.1016/j.jad.2007.01.007

[30] Raison CL, Woolwine BJ, Demetrashvili MF, Borisov AS, Weinreib R, et al (2007) Paroxetine for prevention of depressive symptoms induced by interferon-alpha and ribavirin for hepatitis C. Aliment Pharmacol Ther 25: 1163–74. 17451562 10.1111/j.1365-2036.2007.03316.x

[31] Diez-Quevedo C, Masnou H, Planas R, Castellví P, Giménez D, et al (2011) Prophylactic treatment with escitalopram of pegylated interferon alfa-2a-induced depression in hepatitis C: a 12-week, randomized, double-blind, placebo-controlled trial. J Clin Psychiatry 72: 522–8. 21034680 10.4088/JCP.09m05282blu

[32] De Knegt RJ, Bezemer G, Van Gool AR, Drenth JP, Hansen BE, et al (2011) Randomised clinical trial: escitalopram for the prevention of psychiatric adverse events during treatment with peginterferonalfa-2a and ribavirin for chronic hepatitis C. Aliment Pharmacol Ther 34: 1306–17. 21999489 10.1111/j.1365-2036.2011.04867.x

[33] Morasco BJ, Loftis JM, Indest DW, Ruimy S, Davison JW, et al (2010) Prophylactic antidepressant treatment in patients with hepatitis C on antiviral therapy: a double-blind, placebo-controlled trial. Psychosomatics 51: 401–8. 20833939 10.1176/appi.psy.51.5.401 PMC2994596

[34] Schaefer M, Sarkar R, Knop V, Effenberger S, Friebe A, et al (2012) Escitalopram for the prevention of peginterferon-α2a-associated depression in hepatitis C virus-infected patients without previous psychiatric disease: a randomized trial. Ann Intern Med 157: 94–103. 22801672 10.7326/0003-4819-157-2-201207170-00006

[35] Gleason OC, Fucci JC, Yates WR, Philipsen MA (2007) Preventing relapse of major depression during interferon-alpha therapy for hepatitis C-A pilot study. Dig Dis Sci 52: 2557–63. 17436092 10.1007/s10620-006-9729-5

[36] Kraus MR, Schäfer A, Al-Taie O, Scheurlen M (2005) Prophylactic SSRI during interferon alpha re-therapy in patients with chronic hepatitis C and a history of interferon-induced depression. J Viral Hepat 12: 96–100. 15655055 10.1111/j.1365-2893.2005.00554.x

[37] Moher D, Liberati A, Tetzlaff J, Altman DG (2009) PRISMA Group (2009) Preferred reporting items for systematic reviews and meta-analyses: the PRISMA statement. PLoS Med 6: e1000097. 19621072 10.1371/journal.pmed.1000097 PMC2707599

[38] Jadad AR, Moore RA, Carroll D, Jenkinson C, Reynolds DJ, et al (1996) Assessing the quality of reports of randomized clinical trials: is blindingnecessary? Control Clin Trials 17: 1–12. 8721797 10.1016/0197-2456(95)00134-4

[39] Begg CB, Mazumdar M (1994) Operating characteristics of a rank correlation test for publication bias. Biometrics 50: 1088–101. 7786990

[40] Asnis GM, De La Garza R 2nd (2006) Interferon-induced depression in chronic hepatitis C: a review of its prevalence, risk factors, biology, and treatment approaches. J Clin Gastroenterol 40: 322–35. 16633105 10.1097/01.mcg.0000210099.36500.fe

[41] Trkulja V (2010) Is escitalopram really relevantly superior to citalopram in treatment of major depressive disorder? A meta-analysis of head-to-head randomized trials. Croat Med J 51: 61–73. 20162747 10.3325/cmj.2010.51.61 PMC2829184

[42] Fried MW, Shiffman ML, Reddy KR, Smith C, Marinos G, et al (2002) Peginterferon alfa-2a plus ribavirin for chronic hepatitis C virus infection. N Engl J Med 347: 975–82. 12324553 10.1056/NEJMoa020047

[43] Manns MP, McHutchison JG, Gordon SC, Rustgi VK, Shiffman M, et al (2001) Peginterferon alfa-2b plus ribavirin compared with interferon alfa-2b plus ribavirin for initial treatment of chronic hepatitis C: a randomized trial. Lancet 358: 958–65. 11583749 10.1016/s0140-6736(01)06102-5

[44] Gleason OC, Yates WR, Philipsen MA, Isbell MD, Pollock BG (2004) Plasma levels of citalopram in depressed patients with hepatitis C. Psychosomatics 45: 29–33. 14709758 10.1176/appi.psy.45.1.29

