# Supplementary material for: Can Antidepressants Prevent Pegylated Interferon-α/Ribavirin-Associated Depression in Patients with Chronic Hepatitis C: Meta-Analysis of Randomized, Double-Blind, Placebo-Controlled Trials?
Source: PLoS One. 2013 Oct 30;8(10):e76799. doi: 10.1371/journal.pone.0076799 (PMC3813681; doi:10.1371/journal.pone.0076799)
Supplement: Figure S1 — PRISMA 2009 Flow Diagram. (DOC) [file pone.0076799.s001.doc]

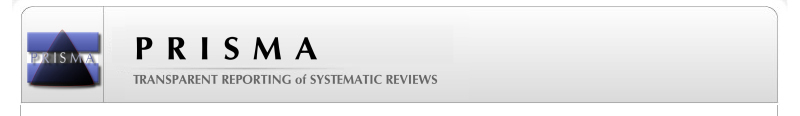
**PRISMA 2009 Flow Diagram**

**Screening**

**Included**

**Eligibility**

**Identification**

Records identified through database searching
(n = 826 )

Additional records identified through other sources
(n =45 )

Records after duplicates removed
(n =391 )

Records screened
(n =391 )

Records excluded
(n = 373 )

Full-text articles assessed for eligibility
(n =18 )

Full-text articles excluded, with reasons

(n = 12)

Non-RCT design (n = 8)

Non-prophylaxis studies (n = 3)

Studies on HCV-HIV co-infection (n = 1)

Studies included in qualitative synthesis
(n =6 )

Studies included in quantitative synthesis (meta-analysis)
(n =6 )
